# Life course socioeconomic determinants of multimorbidity in later life: longitudinal evidence from the Health and Retirement Study

**DOI:** 10.1332/17579597Y2025D000000068

**Published:** 2026-03-09

**Authors:** Mengling Cheng, Eileen M. Crimmins

**Affiliations:** East China University of Science and Technology, China; University of Southern California, USA

**Keywords:** life course models, socioeconomic determinants, multimorbidity, Health and Retirement Study, longitudinal analysis

## Abstract

Using data from the Health and Retirement Study, we examined (a) how childhood socioeconomic status (SES) affects multimorbidity in later life; (b) whether the association between childhood SES and later-life multimorbidity provided empirical support for the critical period model, the sensitive period model, the pathway model and the accumulation model; (c) whether there were cohort differences in the association between childhood SES and later-life multimorbidity. Participants (*N* = 12,601) were grouped into three birth cohorts (1929–38, 1939–45 and post-1945) and followed up from 1998 to 2020. We performed two-level Poisson growth curve models. We found that the association between childhood SES and later-life multimorbidity was modified by SES in adulthood or older age but remained significant, supporting the sensitive period model. Childhood SES affects later-life multimorbidity via SES attainment in adulthood and older age, supporting the pathway model. Persistent disadvantage in childhood, adulthood and older age is a strong risk factor for later-life multimorbidity, supporting the accumulation model. Our results did not support the critical period model. These findings hold among all three cohorts, although to a different extent. Our findings highlight that childhood is a sensitive and malleable period in the life course of an individual when it is possible to break the chain of risk and prevent accumulation of socioeconomic disadvantage in multimorbidity in older age. Moreover, individual lives are embedded in historical and social contexts, the dynamic interplay between which plays a key role in determining the risk of multimorbidity in later life.

## Introduction

The last few decades have witnessed rapid population ageing. As individuals age, they are more susceptible to developing chronic diseases such as cardiovascular conditions, diabetes, and so on (Dogra et al, 2022). Multimorbidity, defined as the co-occurrence of two or more chronic conditions in an individual (Marengoni et al, 2011), is increasingly prevalent globally (Nicholson et al, 2024). Multimorbidity is associated with significant adverse outcomes, including reduced quality of life, elevated healthcare utilisation and increased mortality risk (Xu et al, 2017; Makovski et al, 2019; Chowdhury et al, 2023). Addressing this issue requires a comprehensive understanding of major risk factors and underlying mechanisms.

One of the major risk factors for chronic diseases in later life is low socioeconomic status (SES). Previous studies have found that differences in risk of later-life chronic diseases can be explained by differences in SES in childhood, adulthood or older age (Henchoz et al, 2019; Bof de Andrade et al, 2022; Jungo et al, 2022), through behavioural, psychosocial or material pathways (Fleitas Alfonzo et al, 2022). However, less is understood about the role of life course SES and the underlying patterns (Farina et al, 2023). Life course perspective offers insights into understanding chronic diseases as a continuum that begins early in life and extends into older age (Kuh et al, 2003).

Scholars have proposed four life course models: the critical period model, the sensitive period model, the pathway model and the accumulation model (Ben-Shlomo et al, 2016). The critical period model states that exposures in a specific developmental time window can cause permanent and irreversible change to the structure and/or function of organs, tissues and systems, which is linked to later-life health (Barker, 1997; Bailey et al, 2001; Hallqvist et al, 2004). Outside this time window, there is no excess risk of disease associated with the exposures. A familiar example is the study by Barker and colleagues (1993) where they demonstrated that fetal undernutrition was associated with increased risk of cardiovascular disease in adulthood. As this example illustrates, the critical period model emphasises exposures occurring in developmentally active periods, such as in utero or early childhood when individuals exhibit developmental plasticity (Montez and Hayward, 2011). Critical periods may be more evident for risk of chronic diseases associated with developmental processes within biological subsystems (Ben-Shlomo and Kuh, 2002).

A related but distinct model from the critical period model is the sensitive period model. Like the critical period model, the sensitive period model suggests that exposures during developmentally active periods can have lasting impacts on health in later life. In other words, exposures during a specific developmental window have a pronounced impact on later-life health (Ben-Shlomo and Kuh, 2002). Additionally, it is possible to modify or reverse such influence outside that sensitive time window (Kuh et al, 2003). For example, Haapanen and colleagues (2024) found that the pace of multimorbidity accumulation in later life was programmed early by factors during sensitive developmental periods of gestation, birth, infancy and childhood, particularly by SES during childhood. As illustrated by this example, the sensitive period model highlights the possibility of exposures at later life stages, enhancing or decreasing the influence of early-life exposures.

The pathway model states that health-related exposures occur over the life course and exposures in early life are linked to later-life health through a chain of risks over time (Halfon and Hochstein, 2002). The pathway model emphasises that early-life exposures set in motion lifelong health-related trajectories (Montez and Hayward, 2011). For example, Dekhtyar and colleagues (2019) found that the association between childhood circumstances and multimorbidity accumulation speed was attenuated by subsequent mid- and late-life factors, wherein early-life circumstances influenced later experiences, opportunities and risks. As illustrated by this example, each exposure in a chain of risk can increase the likelihood of subsequent exposures, contributing additively to later health outcomes. Alternatively it may be the temporally proximate adulthood exposures that primarily influence health in later life (Mishra et al, 2010).

The accumulation model states that exposures in early life can have cumulative influences on health outcomes over the life course (Dannefer, 2020). In addition, the accumulation models suggests that adulthood exposures add to, exacerbate or ameliorate earlier exposures (Halfon and Hochstein, 2002). For example, Liu and colleagues (2024) found that the accumulation of more early-life adversity experiences was associated with a higher risk of developing more severe multimorbidity trajectories. The idea behind the accumulation model is similar to the notion of allostatic load, suggesting that as the number, duration and intensity of exposures increase, biological systems experience cumulative damage (Ben-Shlomo and Kuh, 2002).

A cohort refers to a group of individuals who experienced the same events within the same time frame (Ryder, 1965). From a life course perspective, cohorts were shaped by distinct social changes and historical events, which can influence their health (Elder and George, 2016). Consequently, the associations between SES and health may vary across cohorts. Research has demonstrated that the impact of SES tends to be more pronounced in younger cohorts compared to older cohorts (Lynch, 2003; 2006; Yang and Lee, 2009; Morciano et al, 2015). This variation can be attributed to cohort-specific experiences that shaped cohort members’ life conditions over the life course (Ploubidis et al, 2014). Cohort effects typically reflect broader social and historical changes that are exogenous to individual developmental process (Murphy and Yang, 2018) and highlight exposures to risk or protective factors in early life or accumulated over the life course (Kuh et al, 2003). These exposures can have differential impacts on later-life health. Emerging evidence suggests that life course models linking SES to later-life health may operate differently across cohorts (Ploubidis et al, 2014; Sha et al, 2018). For instance, among individuals aged 75 and older, early-life SES has a stronger influence on later-life health compared to adulthood SES and older-age SES. This reflects the long-term impact of the Great Depression of the 1930s, which this cohort experienced during their formative years (Ploubidis et al, 2014). Such findings underscore the importance of considering historical and social contexts when examining the relationship between SES and health across the life course.

Guided by the life course perspective, this study aims to investigate the long-term link between early-life SES and later-life chronic diseases, by formally testing four life course models: the critical period model, the sensitive period model, the pathway model and the accumulation model. This study focuses on three US cohorts: those born during the Great Depression (1929–38), during the Second World War (1939–45) and after the Second World War (post-1945). These cohorts experienced distinct social changes and historical events, such as economic recession, wartime conditions and the postwar expansion of education, offering a unique opportunity to examine how life course models operate across different contexts and cohorts.

## Methods

### Data and sample

This study used 12 waves of data from the Health and Retirement Study (HRS, 1998–2020). The HRS is a longitudinal panel study that surveys a nationally representative sample of about 20,000 older adults above age 50 in the US. Participants were interviewed every two years since 1992. Since its launch, the HRS has collected information about demographics, SES, health, wellbeing, pension plans, healthcare expenditures, and other aspects. Response rates in the HRS core interview are consistently high, ranging from 73.9 per cent to 89.1 per cent between 1998 and 2020 (HRS Staff, 2023). Although longitudinal data is subject to attrition, the HRS employs targeted recruitment and other retention strategies to minimise participant loss (Sonnega et al, 2014).

**Figure 1:**
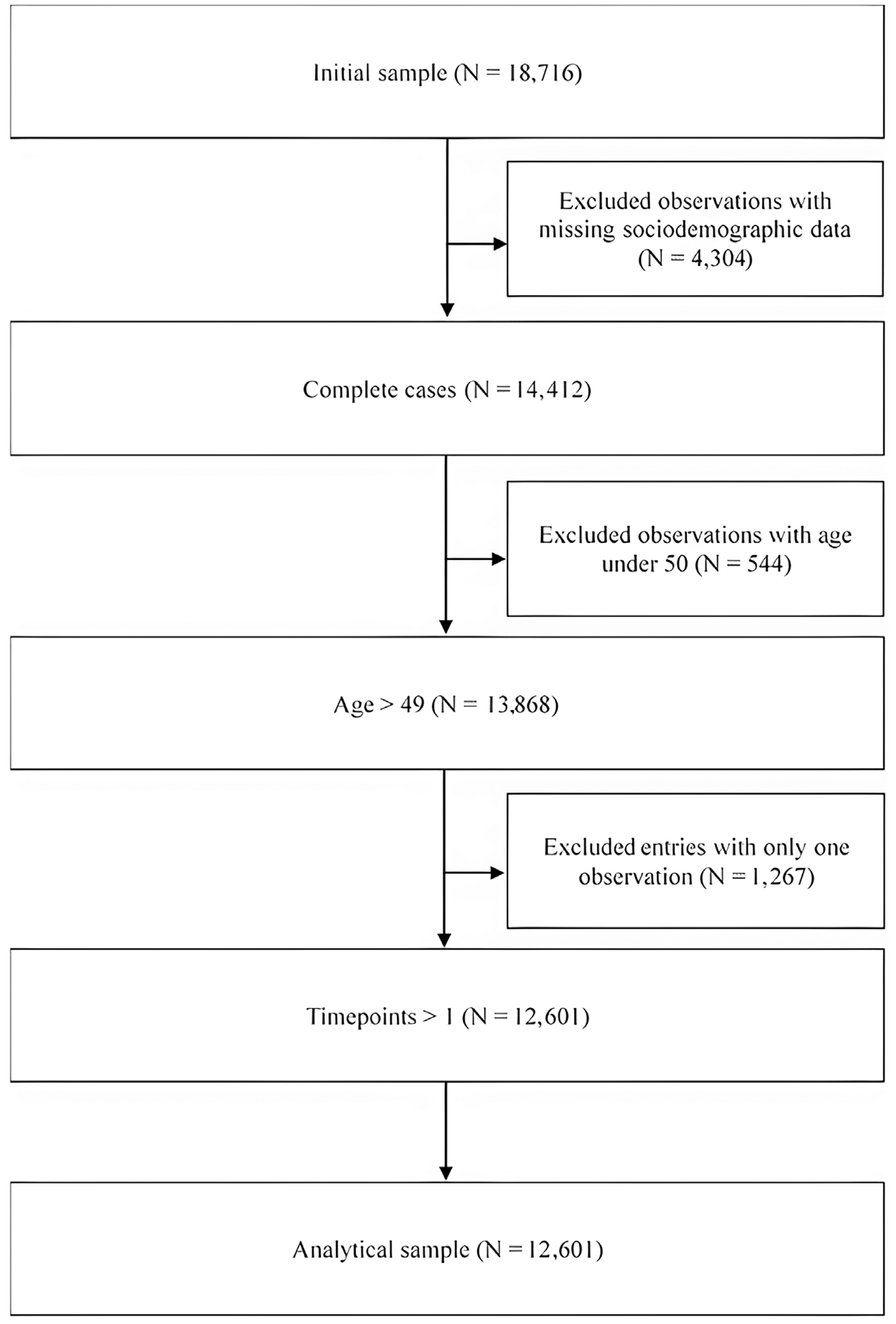
Flow diagram of the analytical sample

To take advantage of the hierarchical structure of the HRS data, we treated wave-specific observations (level-1 units) as nested within participants (level-2 units). Within-participant observations were eligible for inclusion if they met the following three criteria: (a) sociodemographic data were not missing; (b) age at interview was 50 or above; and (c) there were at least two observations. After excluding ineligible observations, our final analytical sample comprised 71,402 observations from 12,601 participants. The flow diagram of the sample is detailed in Figure 1.

### Measures

#### Multimorbidity

Multimorbidity is defined as the presence of two or more coexisting chronic conditions in an individual (Marengoni et al, 2011). In this study multimorbidity was operationalised as a count (≥2) of the following eight chronic conditions available across all HRS waves: hypertension, diabetes, cancer, lung disease, heart disease, stroke, psychiatric disease and arthritis. Each condition was dichotomised (0=no, 1=yes), and individuals with two or more of these eight conditions were classified as having multimorbidity. The count of the number of chronic diseases within each participant ranges from 0 to 8. This is a time-varying variable.

The selection of these eight conditions was justified by two factors: clinical relevance to older US adults and data availability. Specifically, these chronic conditions account for the majority of morbidity and mortality in this population (Fisher et al, 2005). In addition, these chronic conditions were assessed identically across HRS waves, ensuring longitudinal consistency.

#### Life course socioeconomic status

Drawing on theoretical frameworks positing that socioeconomic disadvantage operates through integrated resource deprivation (Conway et al, 2019), we treated SES as a single construct. This enables us to capture multiple dimensions of SES that reflect individuals’ access to social and economic resources. We measured SES in three life course periods: childhood, adulthood and older age. In addition, we assessed cumulative SES over the life course.

Childhood SES was measured by both mother’s highest level of education and father’s highest level of education (1=less than upper secondary, 2=upper secondary/vocational, 3=tertiary), father’s occupation when the participant was age 16 (1=blue collar, 2=military, 3=white collar), and family financial situation before age 16 (1=poor, 2=about average, 3=pretty well off).

Adulthood SES was measured by the participant’s highest level of education (1=less than upper secondary, 2=upper secondary/vocational, 3=tertiary) and longest occupation over the life course (1=blue collar, 2=military, 3=white collar).

Older-age SES was measured by income and wealth. Participants reported their total income and their spouse’s total income in the last calendar year. This study used the imputed income and wealth variables generated and provided by the HRS team as part of their core data products. When components were missing in the source data, the HRS performed the imputations per their standard protocols, detailed in the ‘Income and Wealth Imputations’ documentation. Considering inflation, we adjusted total household income with the annual consumer price index (World Bank, 2021) for each year. To account for the difference between single and coupled participants, we equivalised the inflation-adjusted household income (that is, dividing by the square root of two for coupled participants or by one for single participants; OECD, 2013). To consider the income dynamics of older adults, we treated equivalised income as a time-varying variable and created equivalised income decile for each participant in each wave (from 1=bottom 10 per cent to 10=top 10 per cent). For wealth, we created an inflation-adjusted wealth decile for each participant in each wave (from 1=bottom 10 per cent to 10=top 10 per cent).

Cumulative SES over the life course was measured by an index indicating cumulative advantages or disadvantages (Lyu and Burr, 2016) in SES over childhood, adulthood and older age. First, we created a childhood SES index based on the average of the four childhood SES indicators: mother’s education (1=upper secondary/vocational or above, 0=otherwise), father’s education (1=upper secondary/vocational or above, 0=otherwise), father’s occupation (1=white collar or military, 0=otherwise) and family financial situation (1=average or well-off, 0=otherwise). Then we dichotomised the childhood SES index at the median (1=above median, 0=at or below the median). Second, we generated an adulthood SES index covering education (1=upper secondary/vocational or above, 0=otherwise) and longest occupation (1=white collar or military, 0=otherwise) and dichotomised it at the median (1=above median, 0=at or below the median). Third, following the same procedure, we created an older-age SES index covering income (1=above median, 0=at or below the median) and wealth (1=above median, 0=at or below the median). Lastly, we combined the childhood SES index, adulthood SES index and older-age SES index into a cumulative SES index that ranged from 0 to 3 (from 0=SES disadvantage to 3=SES advantage over the life course).

### Cohorts

In line with previous studies (Sieber et al, 2019), we define three birth cohorts: 1929–38 (that is, born during the Great Depression), 1939–45 (that is, born during the Second World War) and post-1945 (that is, born after the Second World War).

### Covariates

We included the following covariates: age, gender (1=man, 2=woman), race (1=White/Caucasian, 2=Black or African American, 3=other) and marital status (1=unmarried, 2=married), because these are reported to be important confounders of the association of life course SES and later-life multimorbidity (Ploubidis et al, 2014).

### Statistical analysis

To estimate multimorbidity trajectories over time, we built two-level growth curve models. In our models, wave-specific observations (level-1 units) were nested within participants (level-2 units). We chose to use Poisson regression instead of negative binomial regression because the overdispersion test did not reject the null hypothesis of equidispersion, *χ^2^* (2, *N* = 71,402) = 30,485, *p* = 1.00. We chose to use Poisson growth curve models instead of linear growth curve models because the outcome variable, multimorbidity, is a count variable that follows a Poisson distribution (King, 1988). We performed a series of two-level Poisson growth curve models to formally test life course models in various cohorts in the HRS longitudinal sample. Wave-specific weights at respondent level were used to reduce bias.

A formal mediation analysis comprised of three sets of regression models was conducted (Lee et al, 2021). The first model regressed adulthood SES on childhood SES. The second model regressed older-age SES on childhood SES and adulthood SES. The third model regressed multimorbidity on childhood SES, adulthood SES and older-age SES. Full mediation is confirmed if the association between childhood SES and later-life multimorbidity becomes non-significant after accounting for adulthood and older-age SES, indicating these later measures completely explain the relationship. Partial mediation is confirmed if this association persists but is attenuated, signifying adulthood and older-age SES partially explain the association. All models were estimated in R (version 4.4.0).

### Appraisal of life course models

The appraisal of life course models has been inspired by a review of Wagner and colleagues (2022). The critical period model is supported if childhood SES alone is related to multimorbidity in later life and is not attenuated by the adjustment of adulthood or older-age SES. The sensitive period model is supported if childhood SES is associated with multimorbidity in later life and if the association is attenuated but remains significant after adjusting for adulthood or older-age SES. The pathway model is supported if childhood SES is mediated, either partially or fully, by adulthood or older-age SES. The accumulation model is supported if cumulative SES is related to multimorbidity in later life.

## Results

Our analytical sample comprised 71,402 observations from 12,601 participants. Among them, a total of 596 participants were born between 1929 and 1938, a total of 1,064 participants were born between 1939 and 1945, and a total of 10,941 participants were born after 1945. Average follow-up time for these three cohorts were 15.3 years, 17.5 years and 10.5 years, respectively. Table 1 reports sample characteristics and descriptive statistics for predictors and outcome variables used in our models. Individuals of Cohort 1939–45 and Cohort post-1945 had better childhood SES, adulthood SES and older-age SES compared to those of Cohort 1929–38. The percentage of multimorbid individuals and mean number of chronic diseases within each participant was lower among Cohort 1939–45 (46.5 per cent) and Cohort post-1945 (40.2 per cent) compared to those of Cohort 1929–38 (62.9 per cent).

Table 2 summarises associations between childhood SES and multimorbidity in later life across Cohort 1929–38, Cohort 1939–45 and Cohort post-1945. Our results provided consistent support for the sensitive period model, but not for the critical period model. Higher SES in each life course period was associated with lower risk of multimorbidity in later life (IRRs < 0.96, *p* < 0.01). Furthermore, we observed that there was scope of modifying the impact of childhood SES on later-life multimorbidity. The association between childhood SES and later-life multimorbidity was attenuated by adulthood SES or older-age SES but remained significant (IRRs < 0.94, *p* < 0.001). Interestingly, the protection of higher childhood SES against later-life multimorbidity was comparable in younger cohorts of Cohort 1939–45 (IRR = 0.91, *p* < 0.01) and Cohort post-1945 (IRR = 0.92, *p* < 0.001), but particularly pronounced among Cohort 1929–38 (IRR = 0.88, *p* < 0.001).

**Figure 2:**
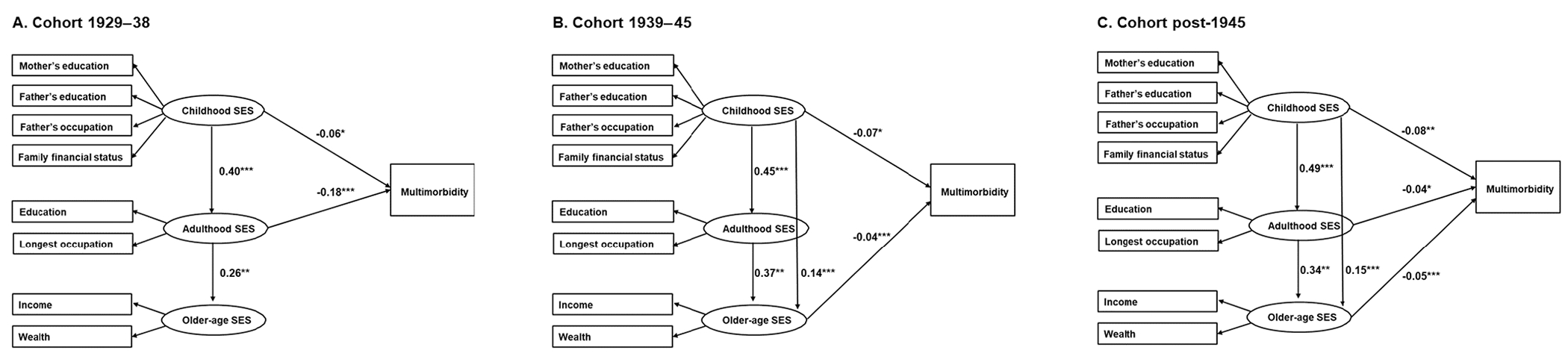
Life course SES and multimorbidity in later life *Note*: For clarity purpose, standardised coefficients estimates are displayed. Non-significant paths were omitted for the purpose of simplicity. All models were adjusted for age, gender, race and marital status. *p < 0.05. **p < 0.01. ***p < 0.001.

Figure 2 shows associations between life course SES and multimorbidity in later life in three cohorts. Our results supported the pathway model. Childhood SES predicted adulthood SES and adulthood SES predicted older-age SES, suggesting a carry-over effect on life course SES. Socioeconomic advantage of the family of origin reduced the risk of multimorbidity in later life by increasing the chances of achieving higher SES in adulthood and older age. Among Cohort 1929–38, individuals with higher childhood SES were more likely to have higher adulthood SES (β = 0.40, *p* < 0.001), which decreased their risk of multimorbidity in later life (β = −0.18, *p* < 0.001). Older-age SES did not affect later-life multimorbidity directly. The mediation effect accounted for 54.5 per cent of the total effect of childhood SES on risk of multimorbidity in later life among this cohort. Among Cohort 1939–45, individuals with higher childhood SES were more likely to have higher adulthood SES (β = 0.45, *p* < 0.001), which increased their chances of achieving higher older-age SES (β = 0.37, *p* < 0.01). Such a chain of protective factors helped to lower the risk of multimorbidity in later life (β = −0.04, *p* < 0.001). The mediation effect accounted for 8.7 per cent of the total effect of childhood SES on risk of multimorbidity in later life among this cohort. Among Cohort post-1945, individuals with higher childhood SES tended to have higher adulthood and older-age SES, both of which protected them against risk of multimorbidity in later life (βs < −0.04, *p* < 0.05). The mediation effect through adulthood SES and older-age SES accounted for 9.4 per cent of the total effect of childhood SES on risk of multimorbidity in later life among this cohort.

Table 3 shows associations between cumulative SES over the life course and multimorbidity in later life in three cohorts. Our analyses showed empirical support for the accumulation model. Findings suggested that compared to individuals who had persistent SES disadvantages in childhood, adulthood and older age, individuals with moderate SES or high SES over the life course tended to have a decreased risk of multimorbidity in later life (IRRs < 0.83, *p* < 0.05). The observed accumulation effect was most pronounced among cohort born after 1945 (IRRs < 0.74, *p* < 0.001).

We conducted two sets of sensitivity analyses to examine how the operationalisation of multimorbidity and SES might influence our main findings. The first set of sensitivity analyses used an alternative definition of multimorbidity: physical-mental multimorbidity. This was defined as the co-occurrence of one or more conditions from the following seven physical conditions (hypertension, diabetes, cancer, lung disease, heart disease, stroke, arthritis) and a chronic mental health condition (psychiatric disease). Findings were consistent with those of the main analyses. Results from this sensitivity analysis are reported in the Supplementary Material (Table S1–2; Figure S1). The second set of sensitivity analyses used different SES measures: education, occupation and income. Findings were consistent with those of the main analyses. Results from this sensitivity analysis are reported in the Supplementary Material (Table S3).

## Discussion

Using the 1998–2020 data from the HRS, we performed Poisson growth curve modelling to examine how SES at three life periods (childhood, adulthood and older age) affects multimorbidity in later life among US older adults. We tested four life course mechanisms linking childhood SES to multimorbidity: the critical period model, the sensitive period model, the pathway model and the accumulation model. We stratified results for Cohort 1929–38, Cohort 1939–45 and Cohort post-1945. Echoing a recent review on empirical evidence regarding life course models and multimorbidity (Wagner et al, 2022), we found empirical support for the sensitive period model, the pathway model and the accumulation model, but not the critical period model. These results hold among all three cohorts, although to a different extent. Findings were confirmed in sensitivity analyses. Our findings emphasise the complex and interrelated processes underlying the long-term influence of early-life SES on multimorbidity over the life course.

The lack of empirical support for the critical period model indicated that the association between childhood SES and later-life multimorbidity is influenced by adulthood SES or older-age SES. This implies that no single life course period entirely determines the risk of multimorbidity in later life. Nevertheless, despite the lack of empirical support for the critical period model, we recommend that future studies continue to consider this model, because it may be applicable in specific contexts. For instance, historical famines during the in utero period or infancy could lead to severe undernutrition, which has been linked to an increased risk of multimorbidity in later life (Cheng et al, 2023). This aligns with empirical evidence that targeted early-life interventions yield lasting health benefits into adulthood and older age (Ford et al, 2018; Kinra et al, 2020; Maguolo et al, 2023).

In line with the sensitive period model, our findings suggest that childhood SES has a long-term but reversible impact on multimorbidity in later life. In other words, the adverse influences of childhood SES disadvantage may be modified by SES in adulthood or later life. Individuals who experienced early-life socioeconomic disadvantage were likely to fare well in terms of multimorbidity if their SES improved in adulthood or later life. This is consistent with previous studies that tested the association between early-life SES and multimorbidity in later life (Tucker-Seeley et al, 2011; Pavela and Latham, 2016). On the one hand, as a sensitive period, early life is particularly susceptible to environmental deprivation but also highly responsive to environmental enrichment, exerting an influence on multimorbidity in older age. On the other hand, socioeconomic circumstances in later life stages also play a role in shaping multimorbidity trajectories over the life course. This highlights the importance of interventions across various life course periods targeting individuals at risk at the right time. It is worth noting that the absence of support for the critical period model in our study does not diminish the significance of early-life SES as a root of later-life multimorbidity. We emphasise that the early-life exposures play a crucial role in shaping unequal life-course trajectories that eventually impact multimorbidity in later life.

Consistent with the pathway model, we found that the association between childhood SES and multimorbidity in later life is partially mediated by SES in later life stages. The influence of childhood SES on later-life multimorbidity is sequentially transmitted through SES at later life stages. Higher childhood SES leads to higher SES in adulthood that is further related to higher SES in older age. In turn, socioeconomic advantage in adulthood and older age is associated with decreased risk of multimorbidity in later life. Our findings are in line with previous research testing the pathway underlying the association between childhood SES and later-life multimorbidity (Dekhtyar et al, 2019; Henchoz et al, 2019). Early-life socioeconomic environment can launch changes in life-long multimorbidity trajectories via adulthood SES and older-age SES. As suggested by the pathway model, the temporally proximate adulthood or older-age exposure plays a key role in influencing multimorbidity in later life (Montez and Hayward, 2011). This highlights the importance of identifying the underlying pathway linking childhood SES and later-life multimorbidity and breaking the chain of risk.

In line with the accumulation model, our findings show that persistent disadvantage across childhood, adulthood and older age is a strong risk factor for multimorbidity in later life, particularly relative to accumulated advantage over the life course. Individuals with lower childhood SES were more likely to be exposed to adversities or stressful life events and had less access to protective resources, which makes them vulnerable to multimorbidity in later life. These results are consistent with previous studies that tested the cumulative effects of life course SES on multimorbidity in later life (Katikireddi et al, 2017; Jungo et al, 2022). The observed pattern emphasises joined effects of socioeconomic disadvantage in childhood, adulthood and older age. It also reflects that socioeconomic disadvantage may be transmitted across generations. The implication is that the consequences of early-life SES on later-life multimorbidity are not entirely offset by SES in later life stages; rather, the lasting impacts of early socioeconomic disadvantage work in tandem with current low SES.

When examining the life course models in Cohort 1929–38, Cohort 1939–45 and Cohort post-1945, several cohort differences were observed. For the sensitive period model, our results showed that the association between childhood SES and multimorbidity in later life was particularly strong among Cohort 1929–38. This is consistent with previous research testing life course models across cohorts (Ploubidis et al, 2014). A possible explanation is that the Great Depression of the 1930s experienced by this cohort in their childhood reinforced the long-term influence of childhood SES on multimorbidity in older age. For the pathway model, our results showed that adulthood SES explained half of the total effects of childhood SES on later-life multimorbidity among Cohort 1929–38. It is not surprising to see this pattern because, as shown in Table 2, adulthood SES had a strong impact on later-life multimorbidity among this cohort. The strong influence of adulthood SES may be explained by educational expansion and economic growth from the end of the Second World War to the early 1970s (Walters and Rubinson, 1983). For the accumulation model, our results showed that the association between cumulative SES over the life course and later-life multimorbidity was stronger in the younger cohort compared to the older cohort. It is likely that in the context of rising income inequality, younger cohorts may experience higher levels of competition in education and employment, where SES plays a more crucial role in accessing health-related resources and services (Workman, 2022). Furthermore, the trend of social welfare retrenchment in the US may make the protection of higher SES and the disadvantages of lower SES become more pronounced among younger cohorts (Hacker, 2004). These observed cohort differences in life course models highlight the dynamic interplay between individual lives and historical and social contexts. The life trajectories of individuals from different cohorts are shaped by the unique historical times and places they experience (Elder and George, 2016), suggesting that the significance and impact of SES may vary across these contexts and cohorts.

The strengths of this study include using large-scale and longitudinal data (17,000+ participants followed up from 1998 to 2020), performing advanced statistical modelling (two-level Poisson growth curve models), conducting formal tests for four life course models (the critical period model, the sensitive period model, the pathway model and the accumulation model), and considering cohort differences in assessing the association between childhood SES and later-life multimorbidity. It is worth noting that older adults with multimorbidity are highly heterogeneous in that they differ in condition severity, combinations, socioeconomic patterning, and the underlying disease pathways.

Several limitations of the current study warrant acknowledgement. First, this study faced limitations of the life course approach. Specifically, residual confounding from factors unmeasured across the entire life span such as complex gene–environment interactions cannot be fully ruled out. Furthermore, the association between early-life exposure and older-age health is shaped by the cohort’s specific historical and socioeconomic context, therefore findings from one cohort may not generalise to others. Second, childhood SES and chronic diseases are self-reported and retrospective, which may be influenced by recall bias. However, previous studies have shown satisfactory validity for self-reported and recall measures of SES and chronic diseases (Lacey et al, 2012; Van der Heyden et al, 2014). Third, diagnosis of multimorbidity tends to be socioeconomically patterned, with systematic underdiagnosis occurring among individuals in more disadvantaged circumstances (Chen et al, 2023). This differential underdiagnosis indicates that the observed associations between SES and multimorbidity may be underestimates of the true effect sizes. Fourth, the measurement of multimorbidity was restricted to eight chronic conditions in the current study, because these conditions were available and comparable across all waves in the HRS. Future research testing life course models in later-life multimorbidity needs to consider a broader range of chronic conditions where data availability permits. Fifth, the analysis presented here did not investigate specific combinations of chronic conditions within the multimorbid population, as this was not the central aim of our study. Future studies building upon our findings could examine how life course SES influences the risk of developing distinct multimorbidity patterns. Sixth, SES was measured at three life stages, which cannot fully capture the variation in SES across the life course. Future studies could explore the association between SES trajectories and later-life multimorbidity. Seventh, we did not include potential interactions between SES indicators across life stages, as this is beyond the focus of current study. Future research on how socioeconomic advantage/disadvantage in various life course periods combines to shape later-life multimorbidity are warranted. Finally, we stratified the results into three broad cohorts: those born during the Great Depression, during the Second World War and after the Second World War. Future studies investigating cohort differences in life course models should explore more granular birth cohorts for a deeper understanding.

## Conclusion

Despite these limitations, this study reveals the mechanisms through which childhood SES operates over the life course to shape multimorbidity in later life. Our findings provided empirical support for the sensitive period model, the pathway model and the accumulation model; we did not find empirical evidence for the critical period model. Childhood is a sensitive and malleable period in the life course of an individual to break chain of risk and prevent accumulation of socioeconomic disadvantage in multimorbidity in older age. Moreover, individual lives are embedded in historical and social contexts, the dynamic interplay between which play a key role in determining the risk of multimorbidity in later life. This study highlights the importance of life course SES and sociohistorical contexts on multimorbidity in later life and alerts policies to address socioeconomic disparities early and consistently throughout the lifespan to mitigate long-term inequalities in multimorbidity.

## Supplementary Material

Supplementary Material

Supplementary material can be found via https://osf.io/fnzpv/overview?view_only=c71399dac5c5452fbe1f1f06e881550f.

## Figures and Tables

**Table 1:** Sample characteristics

	Cohort 1929–38 (N=596)	Cohort 1939–45 (N=1,064)	Cohort post-1945 (N=10,941)
Average follow-up time (years)	15.3	17.5	10.5
Age (Mean [SD])	74.6 (3.7)	63.6 (4.2)	57.6 (3.4)
Men (%)	29.4	59.0	45.2
White (%)	86.1	81.6	64.4
Currently married (%)	74.7	77.8	71.8
Childhood SES			
Mother’s education (%)			
Less than upper secondary	63.9	45.4	36.4
Upper secondary	24.8	38.3	43.2
Tertiary	11.3	16.3	20.4
Father’s education (%)			
Less than upper secondary	68.5	55.6	42.5
Upper secondary	17.6	26.9	33.6
Tertiary	13.9	17.5	23.9
Father’s occupation (%)			
Blue collar	82.1	77.7	70.8
Military	0.7	1.5	2.6
White collar	17.2	20.8	26.6
Family financial situation before age 16 (%)			
Poor	31.2	25.9	23.7
About average	61.2	62.7	65.8
Pretty well off	7.6	11.4	10.5
Adulthood SES			
Education (%)			
Less than upper secondary	19.6	14.4	13.1
Upper secondary	35.1	29.2	26.3
Tertiary	45.3	56.4	60.6
Longest occupation (%)			
Blue collar	63.5	61.2	63.5
Military	0.2	0.4	0.5
White collar	36.3	38.4	36.0
Older-age SES			
Equivalised annual income (in 2010 USD, Mean [SD])	40,755 (36,761)	64,924 (64,105)	66,996 (164,289)
Household wealth (in 2010 USD, Mean [SD])	587,789 (977,017)	605,331 (1,119,994)	408,990 (1,638,722)
Multimorbidity (%)	62.9	46.5	40.2
Number of chronic diseases (Mean [SD])	2.3 (1.2)	1.9 (1.3)	1.6 (1.3)

*Note*: The proportion of older participants with multimorbidity within each cohort was calculated as the number of older adults reporting two or more chronic conditions divided by the total number of cohort respondents.

**Table 2:** Associations between childhood SES and multimorbidity in later life

	Cohort 1929–38		Cohort 1939–45		Cohort post-1945	
	IRRs	95% CI	IRRs	95% CI	IRRs	95% CI
Childhood SES → Multimorbidity	0.88***	0.86–0.90	0.91**	0.89–0.93	0.92***	0.91–0.93
Adulthood SES → Multimorbidity	0.83***	0.82–0.84	0.93**	0.91–0.95	0.94***	0.93–0.95
Older-age SES → Multimorbidity	0.96**	0.94–0.98	0.95***	0.94–0.96	0.95***	0.94–0.96
Childhood SES → Multimorbidity (adjusted for adulthood SES)	0.94***	0.93–0.95	0.95**	0.94–0.96	0.95***	0.93–0.97
Childhood SES → Multimorbidity (adjusted for older-age SES)	0.92***	0.90–0.94	0.93**	0.92–0.94	0.94***	0.93–0.95

*Note*: IRRs refer to Incidence Rate Ratios. All models were adjusted for age, gender, race and marital status.

* p < 0.05.

** *p* < 0.01.

*** *p* < 0.001.

**Table 3:** Associations between cumulative SES and multimorbidity in later life

	Cohort 1929–38		Cohort 1939–45		Cohort post-1945	
	IRRs	95% CI	IRRs	95% CI	IRRs	95% CI
Lowest life course SES	Reference group		Reference group		Reference group	
Lower life course SES	0.90	0.79–1.03	0.97	0.85–1.09	0.86D***	0.79–0.93
Moderate life course SES	0.81**	0.71–0.93	0.81**	0.71–0.92	0.74***	0.68–0.81
High life course SES	0.83*	0.70–0.99	0.75***	0.65–0.87	0.63***	0.57–0.69

*Note*: IRRs refer to Incidence Rate Ratios. Model was adjusted for age, gender, race and marital status.

* *p* < 0.05.

** *p* < 0.01.

*** *p* < 0.001.

## Data Availability

Data supporting this study are available on the HRS website at https://hrsdata.isr.umich.edu/data-products/. Registration required for access.

## Key messages

- The association between childhood socioeconomic status (SES) and later-life multimorbidity supports the sensitive period, the pathway and the accumulation models.
- Childhood SES affects later-life multimorbidity via SES attainment in adulthood and older age.
- Childhood is a malleable period during which to break chain of risk and prevent accumulation of socioeconomic disadvantage in multimorbidity in older age.
